# Fall Prevalence and Associated Risk Factors in the Hospitalised Adult Population: A Crucial Step Towards Improved Hospital Care

**DOI:** 10.7759/cureus.44146

**Published:** 2023-08-26

**Authors:** Imran Khawaja, Shakeel Ahmad Awan, Dr masroor azam, Muhammad Babar, Dr. Taimoor Khan, Muhammad owais Khalil

**Affiliations:** 1 Department of Internal Medicine, Ayub Teaching Hospital, Abbottabad, PAK; 2 Department of Medicine, Chesterfield Royal Hospital NHS Foundation Trust, Chesterfield, GBR; 3 Emergency Medicine, Dianna Princess of Wales Hospital NHS foundation trust, Wales, GBR; 4 Department of Medicine, Heartlands hospital Birmingham UHB NHS foundation trust, Birmingham, GBR; 5 Cardiology, University Hospitals Derby and Burton, NHS Foundation Trust, Derby, GBR; 6 Medicine, University hospital of derby and Burton NHS Foundation trust, Derby, GBR

**Keywords:** adult population, fall risk assessment, risk factors of falls, falls prevention, elderly falls

## Abstract

Background

Falls among the adult population are a major global health concern with severe repercussions for individuals and healthcare systems. The purpose of this study was to investigate the prevalence and associated risk factors of falls in hospitalized patients in order to improve hospital care for elderly adults.

Materials and methods

The research was conducted at two institutions of tertiary care in Abbottabad, Pakistan. After extensive screening and obtaining informed consent, a total of 210 participants aged 50 and older were enrolled in the study. Mental status, history of falls, ambulation/elimination status, vision, gait/balance, systolic blood pressure, medication use, and predisposing diseases were evaluated using the Long Term Care Fall Risk Assessment Form. Additionally, the Dynamic Gait Index was utilized to evaluate various aspects of gait.

Results

58.6% of participants reported a history of falls in the previous year, according to the findings. BMI, imbalance, vertigo, and fear of falling were significantly associated with an increased risk of falls in older individuals. The Long-Term Care Fall Risk Assessment, the Montreal Cognitive Assessment (MoCA), the Dynamic Gait Index (DGI), and the Mini-BESTest scores revealed that patients with a history of falls had inferior functional and cognitive outcomes. Falls were more common among individuals with a robust BMI, especially men.

Conclusions

The study results highlight the multifactorial nature of falls in the adult population and the need for targeted interventions to address modifiable risk factors. To enhance hospital care for high-risk patients, proactive fall prevention strategies, including regular risk assessments and individualized interventions, should be implemented. This study provides important insights into the prevalence and causes of accidents among hospitalized patients, particularly in developing nations such as Pakistan. ​​​​​​

## Introduction

Falls are a significant global health concern, with devastating effects on individuals and healthcare systems. Estimates indicate that falls were the 13th leading cause of death globally in 2015. Between 1990 and 2015, fall-related fatalities increased by a startling 55%, reaching approximately 540,000 per year [1]. Falls and fall-related injuries are significant clinical and public health concerns among patients, resulting in severe physical injuries and causing social, economic, and psychological effects such as depression, anxiety, and diminished confidence [2-5].

The elderly population is especially susceptible to fall-related injuries and fatalities, which raises important public health concerns. This age group is at increased risk for chronic pain, disability, functional impairments, and death [6]. Moreover, as the global elderly population grows, accidents are becoming a significant global health concern [7]. According to the Centres for Disease Control and Prevention (CDC), falls are the leading cause of injury-related death among the elderly. Injuries resulting from falls are the leading cause of disability and death in the elderly population. Globally, 424,000 falls occur annually, with 37.3 million requiring medical attention, resulting in 12% to 24% of all visits to the emergency room [6,8].

Over 82% of fall-related deaths and 92% of disability-adjusted life years (DALY) losses occur in low- and middle-income countries [9]. In these regions, hospital settings are of utmost importance, as inpatient departments are the primary and most frequent entry point for the geriatric into healthcare [6]. Three million elderly hospitalized patients visited the emergency department for fall-related injuries in 2019. As life expectancy continues to increase, the number of individuals over the age of 65 is expected to rise globally, and countries such as Pakistan anticipate a surge in their geriatric population from 12 to 13 million to 18 million by 2050 [9]. The National Injury Survey in Pakistan, for example, reports an annual incidence rate of 8.85 fall-related injuries per 1,000 people [9].

More than 400 modifiable risk factors for falls have been identified [10,11]. Age-related chronic conditions (e.g., Parkinson's disease, stroke, degenerative joint disease) increase fall risk through stiffness, instability, and weakening musculature in the elderly population. Other contributors to fall risk include disorientation, cognitive impairment, urinary incontinence, melancholy, postural hypotension, and visual impairments. In addition, certain medications pose a significant risk of falling for the elderly [12].

In addition, fear of falling can lead to poor posture, decreased walking speed, and muscle frailty, while medical factors such as a history of falls or care dependency can increase the risk of falling. In addition to the use of assistive devices, environmental factors such as low illumination, slippery floors, and inadequately fitting footwear or apparel also contribute to accidents among older inpatients. In addition, characteristics of the care staff and the care milieu may play a role in the occurrence of accidents among geriatric inpatients [13].

Accidents involving this population are a pressing concern for hospital wards that house adult patients, including surgical, neurological, and medical wards. It is of the uttermost importance to ensure patient safety, making fall prevention and the creation of a secure environment fundamental rights that ensure high-quality treatment. Risk assessment is essential for identifying and managing fall risk factors, allowing healthcare professionals to prevent falls and their negative outcomes [14].

However, despite the significance of falls in the older adult population, few studies have explored the prevalence, factors, and determinants of falls among the elderly in hospitals, particularly in developing nations like Pakistan. Thus, this study aims to examine the prevalence and antecedents of falls in adult hospitalized patients, contributing valuable insights for planning measures to prevent accidents among the elderly and enhance their physical and mental health.

## Materials and methods

Study setting and population

This cross-sectional research was conducted at two tertiary care institutions in the Pakistani city of Abbottabad. Inclusion criteria encompassed men and women aged 50 years and older who were currently admitted to hospitals due to various medical conditions. Participants were required to possess the capability of moving indoors autonomously or with the assistance of mobility aids. Conversely, exclusion criteria comprised individuals who faced significant mobility challenges, even with the utilization of assistive devices, and those with severe medical conditions that impeded their mobility. Additionally, individuals currently undergoing physiotherapy or physical fitness training were excluded. These criteria were thoughtfully designed to create a diverse and representative participant pool, ensuring that the study's findings would offer valuable insights into the prevalence of falls and the underlying risk factors within the geriatric population. By excluding participants undergoing interventions that could potentially influence mobility and fall risk, the study aimed to achieve a focused investigation, specifically within the context of a hospital setting.

Study sample

A rigorous sample selection process was undertaken following the STROBE (Strengthening the Reporting of Observational Studies in Epidemiology) criteria. The study initially identified a potential pool of 310 participants consisting of men and women aged 50 years and older who were admitted to two tertiary care hospitals in Abbottabad, Pakistan. From this pool, 210 eligible participants were ultimately enrolled in the study. The selection process involved meticulous screening to ensure participants met the predefined inclusion and exclusion criteria. Participants who met the age requirement, were hospitalized for various medical conditions, and were capable of indoor mobility, either independently or with mobility assistance, were included. Conversely, individuals with severe medical conditions that substantially hindered mobility, those unable to walk even with assistive devices, and those undergoing physiotherapy or physical fitness training were excluded. Each eligible participant provided informed consent before participating in the study, ensuring ethical standards and respecting their autonomy.

Data collection

The data were collected in the hospital units where the admitted patients were located. All participants were administered a comprehensive questionnaire in the local language. The questionnaire included a history of any previous falls, sociodemographic information such as age, height, weight, income, educational qualifications, and marital status, as well as the medical history of the participant. In addition, the questionnaire inquired about specific risk factors for falls, such as the use of a cane or walker, falls within the past three months, the presence of acute or chronic illnesses, the types and numbers of medications being taken, and physical deficits, such as gait and balance disorders, deficits, arthritis-related pain, visual and auditory impairments, epilepsy, parkinsonism, vertigo, syncope, dizziness upon standing, foot problems, and difficulty walking.

Subjects were instructed to complete the survey responses, and the researcher provided assistance as needed to ensure accurate and comprehensive responses.

Assessment tools

Four distinct validated evaluation instruments were used to evaluate functional and cognitive outcomes: the Long-Term Care Fall Risk Assessment consisted of eight components, including the participant's mental status, history of falls within the previous three months, ambulation and elimination status, vision, gait and balance, systolic blood pressure, medications, and predisposing diseases. The participants were divided into low-risk (total score of 10) and high-risk (total score >10) categories based on the total score [15]. Montreal Cognitive Assessment (MoCA) scores range from 0 to 30, with 26 or higher being considered normal [16]. The Dynamic Gait Index (DGI) is commonly used to assess the fall risk of senior individuals. A score of 19 or less indicates that falls are more likely to occur in this population. The DGI is administered to individuals using assistive devices [17] and evaluates eight distinct aspects of locomotion. The Mini-BESTest assesses dynamic balance, functional mobility, and locomotion. It consists of 14 items, each of which carries a maximum score of 2. The cumulative result on the Mini-BESTest is 28, and the minimum passing score is 16. A score below 16 is regarded as inadequate and indicative of a higher risk of collapsing [18].

Data analysis

Version 24.0 of SPSS (IBM Corp., Armonk, NY) was utilized for data analysis. We utilized frequency distributions and descriptive statistics to characterize the study sample. The chi-square test was used to examine the association between the risk of stumbling and various risk factors. In addition, continuous variables, such as the degree of functional impairment between the high-risk and low-risk categories, were analyzed using the chi-square test.

## Results

The total sample for the study was 210 patients with an age range of 51-75 (mean = 59.77 ± 4.762). Among participants, 123 (58.6%) reported a history of falls in the previous year, with a greater percentage of males at 78 (63.4%). Only 38 (18.1%) had undergone any fall assessment in their lives. Demographic and clinical characteristics are shown in Table 1.

**Table 1:** Demographic and clinical characteristics

Variable	N	%
Gender		
Male	133	63.3
Female	77	36.7
Educational attainment		
No schooling	101	48.1
Primary	58	27.6
Secondary and above	51	24.3
BMI		
Underweight (<18.5)	33	15.7
Normal (18.5–24.9)	129	61.4
Overweight (25.0–29.9)	44	21.0
Obese (>30)	4	1.9
History of fall		
Yes	123	58.6
No	87	41.4
Ever undergone fall assessment		
Yes	38	18.1
No	172	81.9

Risk factors associated with fall accidents are shown in both groups (group 1 = patients with H/O falls, group 2 = patients without H/O falls). BMI, imbalance, vertigo, and fear of falling were statistically associated with the risk of falls among adults (Table 2).

**Table 2:** Association of risk factors with a history of falls Chi square test, *P<0.05

Risk factor	Patients with H/O falls		Patients without H/O falls		P-value
	N	%	N	%	
BMI					
Underweight (<18.5)	26	21.1	7	8.0	0.04*
Healthy (18.5–24.9)	69	56.1	60	69.0	
Overweight (25.0–29.9)	25	20.3	19	21.8	
Obese (>30)	3	2.4	1	1.1	
Postural hypotension	75	61.0	51	58.6	0.73
Poor vision	69	56.1	57	65.5	0.17
Chronic conditions	76	61.8	56	64.4	0.70
Walking aid	80	65.0	50	57.5	0.26
imbalance	83	67.5	46	52.9	0.03*
Vertigo	32	26.0	14	16.1	0.04*
Fear of falling	77	62.6	51	58.6	0.04*
Hearing impairment	82	66.7	58	66.7	1.00
Acute medical issue	85	69.1	50	57.5	0.08
Medicine usage	79	64.2	60	69.0	0.45

There was a significant difference between the two groups for functional and cognitive outcomes: Long-Term Fall Risk Assessment (P = 0.00); MoCA (P = 0.00); Dynamic Gait Index (P = 0.00); Mini-BESTest (P = 0.00) (Table 3).

**Table 3:** Comparison of outcome measures among patients with or without a history of falls *P<0.05 DGI: Dynamic Gait Index, MoCA: Montreal Cognitive Assessment

Scale score	Patients with H/O falls		Patients without H/O falls		P-value
	N	%	N	%	
Long-Term Care Fall Risk Assessment					
High risk	117	95.1	11	12.6	0.00*
Low risk	6	4.9	76	87.4	
MoCA					
Impaired cognition	107	87.0	17	19.5	0.01*
Normal cognition	16	13.0	70	80.5	
DGI					
Severe impairment	113	91.9	18	20.7	0.00*
Mild impairment	10	8.1	69	79.3	
Mini-BESTest					
Poor score	111	90.2	20	23.0	0.00*
Good score	12	9.8	67	77.0	

Table 4 presents a comparison of fall accidents among different BMI categories and genders. For the male group, 32.1% (25) of fall accidents occurred in individuals classified as underweight. For the female group, only 2.2% (1 case) of fall accidents occurred in individuals classified as underweight. The majority of fall accidents, 64.4% (29 cases), occurred in individuals classified as having a healthy BMI. 26.7% (12) of fall accidents occurred in individuals classified as overweight. A smaller proportion, 6.7% (three cases), of fall accidents were observed in individuals classified as obese. However, no fall accidents were reported in the obese category for males, which may need further investigation. The observed p-values for the comparison of fall accidents across BMI categories indicate statistical significance for both male and female participants, suggesting that BMI categories play a role in fall occurrences. However, it is important to note that while the association is statistically significant, the clinical significance of these findings should be interpreted in the context of other contributing factors and the overall study objectives. Further analyses and investigations are recommended to better understand the interplay between BMI and fall accidents in adults.

**Table 4:** Comparison of fall accidents by body mass index and gender

	Underweight		Healthy		Overweight		Obese		P-value
	N	%	N	%	N	%	N	%	
Male	25	32.1	40	51.3	13	16.7	0		0.00*
Female	1	2.2	29	64.4	12	26.7	3	6.7	
P-value	0.02*		0.04*		0.06		0.2*		

## Discussion

The purpose of the study was to determine the prevalence and risk factors of falls among adults. The investigation included 210 patients between the ages of 51 and 75, with a mean age of 59.774.762. About 58.6% of participants reported a history of falls in the previous year, whereas only 18.1% had undergone a lifetime fall assessment. The results indicate that males had a higher incidence of stumbling than women. The current study's findings contradicted previous findings that more women than men had a history of stumbling [19,20,21].

Analyses of fall-related risk factors were performed on both patients with and without a history of falls. Adults with a higher BMI, imbalance, vertigo, and fear of stumbling are statistically more likely to stumble. Specifically, individuals with a history of falls were more likely than those without a history of falls to experience a fall accident (21.1% vs. 8.0%). Similar to the other group, the group with a history of accidents had a higher prevalence of imbalance and vertigo. The strongest associations were for a history of accidents, mobility problems, the use of walking aids, vertigo, neurological disorders, and the use of antiepileptic medications [22]. In a comprehensive review of factors and prevention strategies for falls among the elderly in India, it was determined that reduced strength, poor balance, poor vision, decreased cognition, and chronic diseases, as well as a history of falls, were the most significant risk factors for falling. "Bathroom" was reported as the most common location for falls, followed by "road" [23]. Several other psychological factors, such as depression, dementia, feelings of remorse, or low mood due to urinary incontinence, were identified in a separate study [24].

The study also assessed the cognitive and functional outcomes associated with fall accidents. Several outcome measures revealed significant differences between the two groups. Patients with a history of falls had higher rates of long-term fall risk (95.1% vs. 12.6%), impaired cognition (87.0% vs. 19.5%), severe impairment in dynamic locomotion (91.9% vs. 20.7%), and poor scores on the Mini-BESTest for balance (90.2% vs. 23.7%). These results suggest that falls were associated with diminished cognitive and functional outcomes in the study population. Several other studies have investigated the relationship between fall accidents and the cognitive and functional outcomes of various populations. Tinetti et al. [25] discovered that individuals with a history of falls had lower scores on tests of functional mobility and balance, highlighting the association between fall accidents and diminished functional outcomes in the elderly. Montero-Odasso et al. found a higher prevalence of cognitive impairment among individuals with a history of falls, highlighting the association between fall accidents and cognitive impairment in senior adults [26]. Similarly, McAdams-Demarco et al. reported more severe impairment in gait and balance among individuals with a history of falls, supporting the notion that fall accidents are associated with deteriorated gait outcomes in the elderly [27]. Delbaere et al. [28] used the Mini-BESTest to evaluate balance and found that individuals who had experienced falls had substantially lower balance scores, confirming the correlation between fall accidents and poor balance outcomes. In addition, a comprehensive meta-analysis of fall prevention strategies in elderly adults revealed that fall prevention interventions led to a reduction in fall rates and enhanced functional outcomes, such as mobility and balance. These findings are consistent with the findings of the present study, which indicate that fall-related injuries are associated with poorer functional and cognitive outcomes in the adult population, especially among the elderly. Therefore, addressing fall incidents through prevention strategies is essential for improving the overall health and quality of life of this population [29].

The analysis of the data included a comparison of fall-related incidents across various BMI categories and genders. The findings revealed that the majority of fall-related incidents occurred in both male and female individuals with a healthy BMI. Notably, male underweight individuals had a significantly higher incidence of fall incidents (32.1%) than female underweight individuals (2%). Intriguingly, no males in the obese category reported falling, whereas obese women reported a history of falls. Consistent with a prior study by Yi et al., which demonstrated that obesity was associated with a higher risk of recurrent falls in women but that being underweight appeared to be associated with a larger risk of falls in men [30], these results indicate that obesity is associated with a higher risk of recurrent falls in women.

Overall, the study disclosed significant information regarding the risk factors and outcomes associated with adult fall accidents. The results highlighted the importance of BMI, imbalance, vertigo, and fear of falling as risk factors, as well as the influence of fall incidents on the participants' functional and cognitive health. The findings could be used to develop targeted fall prevention strategies and interventions to enhance the safety and well-being of senior adults. While this study provides valuable insights into fall prevalence and associated risk factors among the geriatric population, several limitations should be acknowledged. First, the cross-sectional design of the research restricts our ability to establish causal relationships between the identified risk factors and fall occurrences. Longitudinal studies could offer more comprehensive insights into the temporal aspects of fall risk factors. Second, the study's reliance on self-reported data for certain variables, such as medical history and falls, introduces the possibility of recall bias. Participants might inaccurately recall past events or medical conditions, potentially affecting the accuracy of associations. Lastly, the lack of a control or comparison group could be a limitation of the current study.

## Conclusions

Falls among the adult population are a major global health concern with severe repercussions for individuals and healthcare systems. Over the years, the incidence of fall-related fatalities has increased dramatically, making it imperative to identify and address the associated risk factors. This study aimed to determine the prevalence and risk factors of accidents among hospitalized adult patients. This study revealed that falls are a prevalent problem among senior individuals, with a high proportion of participants reporting a history of falls within the previous year. This emphasizes the need for proactive accident prevention measures and risk assessment protocols in hospital settings, particularly in view of the anticipated increase in the geriatric population. Several modifiable risk factors, including BMI, imbalance, vertigo, and anxiety about falling, were identified as being associated with an increased risk of falls in the elderly population. These findings highlight the significance of interventions tailored to target these risk factors in order to reduce fall-related accidents. In addition, the study revealed a significant association between fall accidents and diminished cognitive and functional outcomes. Improving functional mobility and cognitive health in elderly adults requires the implementation of fall-prevention strategies. The implementation of comprehensive fall prevention programmes is essential for enhancing the quality of hospital care for adult patients. These programmes should include routine risk assessment, the identification of modifiable risk factors, and individualized interventions to reduce falls and their associated consequences. By addressing fall risk factors and implementing effective preventive measures, healthcare systems can take an important step towards providing improved care and ensuring the safety and health of the adult population.
